# Solvability and stability of a fractional dynamical system of the growth of COVID-19 with approximate solution by fractional Chebyshev polynomials

**DOI:** 10.1186/s13662-020-02791-x

**Published:** 2020-07-08

**Authors:** Samir B. Hadid, Rabha W. Ibrahim, Dania Altulea, Shaher Momani

**Affiliations:** 1 Department of Mathematics and Sciences, College of Humanities and Sciences, Ajman University, Ajman, UAE; 2 Informetrics Research Group, Ton Duc Thang University, Ho Chi Minh City, Vietnam; 3 Faculty of Mathematics & Statistics, Ton Duc Thang University, Ho Chi Minh City, Vietnam; 4 Faculty of Science, University of Groningen, Groningen, The Netherlands; 5 Department of Mathematics, Faculty of Science, University of Jordan, Amman, 11942 Jordan

**Keywords:** Conformable calculus, Fractional calculus, Fractional differential operator, Fractional integral operator, Dynamic system, COVID-19

## Abstract

Lately, many studies were offered to introduce the population dynamics of COVID-19. In this investigation, we extend different physical conditions of the growth by employing fractional calculus. We study a system of coupled differential equations, which describes the dynamics of the infection spreading between infected and asymptomatic styles. The healthy population properties are measured due to the social meeting. The result is associated with a macroscopic law for the population. This dynamic system is appropriate to describe the performance of growth rate of the infection and to verify if its control is appropriately employed. A unique solution, under self-mapping possessions, is investigated. Approximate solutions are presented by utilizing fractional integral of Chebyshev polynomials. Our methodology is based on the Atangana–Baleanu calculus, which provides various activity results in the simulation. We tested the suggested system by using live data. We found positive action in the graphs.

## Introduction

Coronavirus disease COVID-19 is an infectious disease caused by a newly discovered coronavirus. It has been diffusing quickly in the world and the World Health Organization (WHO) characterized it as a pandemic. The first WHO indication of dyed-in-the-wool situations of COVID-19 was labeled on January 21, 2020 with 282 recognized cases, which is developed with the most present certificate on March 18, 2020, which extends to 191,127 concluded cases (see [1, 2]). Normal growth approaches have been tested to describe the time development of the COVID-19 infection [3]. Fundamentally, by applying the system

$$\begin{aligned} \frac{d}{dt} \varphi (t) =\varphi (t),\quad t \in [0,\infty ), \end{aligned}$$

where *φ* denotes the sum of infected people and the spreading phase, the increasing number of asymptomatic infected individuals was labeled. Recently, an extensive presentation of the fractal-fractional dynamic system of COVID-19 spread was introduced by Atangana [4].

The current investigation concerns the fractional dynamic system of the growth laws by exploiting the idea of fractional calculus. This idea contains an important term, which is the exponential law to find and accept the graph of the growth. The existence and uniqueness consequences are deliberated in the application of the fixed-point theory of self-mappings. Other properties are observed, such as the approximate solvability using the fractional Chebyshev polynomials.

## Fractional dynamic system (FDS)

In this section, we construct the dynamic system of coupled equations. Before that, we need the following preliminaries about the conformable calculus.

### Atangana–Baleanu calculus (ABC)

In recent decades, several physical complications have been displayed when employing the fractional calculus. The fundamental clarifications for applying fractional derivative illustrations are that various arrangements, constructions, and inequalities show the ability to remember past, or nonlocal properties, which cannot be stimulated using normal order derivatives. The fundamental ideas and applications of fractional calculus and fractional differential equations can now be found in many surveys. While most of the principal studies were based on the procedure of the Riemann–Liouville fractional order derivative, or the Caputo fractional order derivative, it has been observed that these derivatives have the property that their kernels have a singularity at the end of the interval of interest. The essential differences among the arbitrary derivatives are their unlike kernels which can be selected to fit the requirements of different applications. For example, the central variations between the Caputo fractional derivative, the Caputo–Fabrizio derivative [5], and others are that the Caputo calculus is expressed by employing a power law, while the Caputo–Fabrizio derivative is characterized by using an exponential decay. Atangana–Baleanu operator is introduced by suggesting the generalized Mittag-Leffler function [6].

#### Definition 1

(Fractional differential operator)

A differential operator $\Delta ^{\nu }, \nu \in (0,1)$ is called a fractional Atangana–Baleanu derivative of order *ν* of a function *φ* if and only if $\Delta ^{\nu }$ can be written as

$$\begin{aligned} \Delta ^{\nu } \varphi (t)= \frac{1}{1-\nu } \int _{0}^{t} \varphi '( \tau ) \varXi _{\nu } \biggl(\frac{-\nu }{1-\nu } (t-\tau )^{\nu } \biggr) \,d \tau,\quad t\geq 0, \end{aligned}$$

where *Ξ* indicates the Mittag-Leffler function. The fractional integral is formulated by

$$\begin{aligned} J^{\nu }\varphi (t)= (1-\nu ) \varphi (t)+\frac{\nu }{\varGamma (\nu )} \int _{0}^{t}\varphi (\tau ) (t-\tau )^{\nu -1} \,d\tau. \end{aligned}$$

### Construction of FDS

In the construction of FDS, we denote by $\varXi (t) $ the increasing overall number of infected persons, which is the sum of the number of the increasing recognized infected individuals $\varphi (t)$ and of the asymptomatic transmissions $\psi (t)$, that is, $\varXi (t) =\varphi (t) + \psi (t)$. Regarding the statistics of $\varphi (t)$, the number of transitions on and off, and cured people are convoluted, because they have been previously ill. Therefore, there are rate functions linking *φ* and *ψ*. We formulate the coupled-FDS as follows:

1 $$\begin{aligned} \begin{aligned} &\Delta ^{\nu }\varphi (t) =\alpha _{1}(t) \varphi (t)+ \alpha (t) \psi (t), \\ & \Delta ^{\nu }\psi (t) =\beta _{1}(t) \psi (t)+\beta (t) \varphi (t), \end{aligned} \end{aligned}$$

where $\alpha,\alpha _{1}$ and $\beta, \beta _{1}$ are the connection rate continuous functions of *ψ* in $\Delta ^{\mu }\varphi (t)$ and *φ* in $\Delta ^{\nu } \psi (t)$, respectively. They describe the damping properties in line for the control energy.

## Results

In this section, we proceed to discuss the solution existence and uniqueness for system (1). Moreover, we investigate the controller solution from different views.

### Stability of solution

In this section, we deal with the stability of the unique solution via fixed point theorem. System (1) can be expressed by the general system

2 $$\begin{aligned} \begin{aligned} &\Delta ^{\nu }\varphi (t) =X(t, \varphi,\psi ), \\ & \Delta ^{\nu }\psi (t) =A(t,\varphi,\psi ), \end{aligned} \end{aligned}$$

satisfying the following hypotheses:

1. Assume that $X: [0,T]\times \mathbb{R}\times \mathbb{R} \rightarrow \mathbb{R}$ is a nondecreasing continuously differentiable function with $X (0,0,0) =0$ and nonvanishing in a compact interval $(0, T] $. Furthermore, there is a positive constant *κ* such that
  $$\begin{aligned} \bigl\vert X(t,\varphi _{1},\psi _{1})-X(t,\varphi _{2},\psi _{2}) \bigr\vert \leq \kappa \bigl( \vert \varphi _{1}-\varphi _{2} \vert + \vert \psi _{1} -\psi _{2} \vert \bigr). \end{aligned}$$
2. Assume that $A: [0,T]\times \mathbb{R}\times \mathbb{R} \rightarrow \mathbb{R}$ is a nondecreasing continuously differentiable function with $A (0,0,0) =0$ and nonvanishing in a compact interval $(0, T]$. In addition, assume that there exists a positive constant *K* such that
  $$\begin{aligned} \bigl\vert A(t,\varphi _{1},\psi _{1})-A(t,\varphi _{2},\psi _{2}) \bigr\vert \leq K \bigl( \vert \varphi _{1}-\varphi _{2} \vert + \vert \psi _{1}- \psi _{2} \vert \bigr). \end{aligned}$$

We aim to establish the existence and uniqueness of solution to system (2) using self-mapping fixed point theorem [7].

#### Lemma 3.1

*Let*$(\aleph,\vartriangle )$*be a complete metric space and*$\mathcal{U}\colon \aleph \to \aleph $*a self*-*mapping satisfying the relation*

3 $$\begin{aligned} \flat \bigl(\vartriangle \bigl(\mathcal{U}(\chi ), \mathcal{U}(\eta )\bigr) \bigr)\leq \flat \bigl(\vartriangle (\chi,\eta )\bigr)- \wp \bigl( \vartriangle (\chi,\eta ) \bigr) \end{aligned}$$

*for all*$\chi,\eta \in \aleph $, *where*$\flat,\wp \colon [0,\infty )\to [0,\infty )$*are both continuous and nondecreasing functions with*$\flat (0)=\wp (0)=0$. *Then*$\mathcal{U}$*admits a unique fixed point*.

Put $\aleph =\mathbb{R}$ and define an operator $\mathcal{P}: \mathbb{R} \times \mathbb{R} \rightarrow \mathbb{R} \times \mathbb{R}$ as follows:

4 $$\begin{aligned} \bigl(\mathcal{P}(\varphi,\psi ) \bigr) (t)= {}&\bigl(\mathcal{P}_{1}( \varphi, \psi ), \mathcal{P}_{2}( \varphi,\psi ) \bigr) (t) \\ ={}& \biggl( (1-\nu ) X(t,\varphi,\psi )+ \frac{\nu }{\varGamma (\nu )} \int _{0}^{t}X(\tau,\varphi,\psi ) (t- \tau )^{\nu -1} \,d\tau, \\ & (1-\nu ) A(t,\varphi,\psi )+ \frac{\nu }{\varGamma (\nu )} \int _{0}^{t} A(\tau,\varphi,\psi ) (t- \tau )^{\nu -1} \,d\tau \biggr). \end{aligned}$$

Since $(\varphi,\psi ) \in \mathbb{R}\times \mathbb{R,}$$\mathcal{P}$ is a self-mapping.

#### Lemma 3.2

*Let the functions*$\mathfrak{B}: \mathbb{R}^{3} \rightarrow \mathbb{R}^{+}$*be defined as follows*:

$$\begin{aligned} \mathfrak{B} \bigl((\varphi _{1},\psi _{1}),(\varphi _{2}, \psi _{2}),( \varphi _{3},\psi _{3}) \bigr)= \max \bigl\{ \vert \varphi _{\imath }-\varphi _{\jmath } \vert + \vert \psi _{\imath }-\psi _{\jmath } \vert :\imath,\jmath =1,2,3, \imath \neq \jmath \bigr\} . \end{aligned}$$

*Then the function*$\mathfrak{B} \in \mathbb{R} $*forms a metric*.

#### Proof

Clearly, $\mathfrak{B}(0)=0$. Furthermore, we have

5 $$\begin{aligned} & \mathfrak{B}\bigl((\varphi _{1},\psi _{1}),(\varphi _{1}, \psi _{1}),(\varphi _{i},\psi _{i})\bigr)+\mathfrak{B}\bigl((\varphi _{2},\psi _{2}),( \varphi _{2},\psi _{2}),(\varphi _{j},\psi _{j})\bigr) \\ &\qquad{}+ \mathfrak{B}\bigl(( \varphi _{3},\psi _{3}),(\varphi _{2},\psi _{2}),(\varphi _{k},\psi _{k})\bigr) \\ &\quad= \max_{i=2,3}\bigl\{ \vert \varphi _{1}-\varphi _{i} \vert + \vert \psi _{1}-\psi _{i} \vert \bigr\} + \max_{j=1,3}\bigl\{ \vert \varphi _{2}-\varphi _{j} \vert + \vert \psi _{2}- \psi _{j} \vert \bigr\} \\ &\qquad{}+ \max_{k=1,2}\bigl\{ \vert \varphi _{3}-\varphi _{k} \vert + \vert \psi _{3}-\psi _{k} \vert \bigr\} \\ &\quad= \max \bigl\{ \vert \varphi _{1}-\varphi _{2} \vert + \vert \psi _{1}-\psi _{2} \vert , \vert \varphi _{1}-\varphi _{3} \vert + \vert \psi _{1}- \psi _{3} \vert \bigr\} \\ &\qquad{} + \max \bigl\{ \vert \varphi _{2}-\varphi _{1} \vert + \vert \psi _{2}-\psi _{1} \vert , \vert \varphi _{2}-\varphi _{3} \vert + | \psi _{2}-\psi _{3}\bigr\} \\ &\qquad{} + \max \bigl\{ \vert \varphi _{3}-\varphi _{1} \vert + \vert \psi _{3}-\psi _{1} \vert , \vert \varphi _{3}-\varphi _{2} \vert + \vert \psi _{3}-\psi _{2} \vert \bigr\} \\ &\quad= 2 \max \bigl\{ \vert \varphi _{1}-\varphi _{2} \vert + \vert \psi _{1}-\psi _{2} \vert , \vert \varphi _{2}-\varphi _{3} \vert + \vert \psi _{2}-\psi _{3} \vert , \vert \varphi _{3}- \varphi _{1} \vert + \vert \psi _{3}-\psi _{1} \vert \bigr\} \\ &\quad> \max \bigl\{ \vert \varphi _{1}-\varphi _{2} \vert + \vert \psi _{1}-\psi _{2} \vert , \vert \varphi _{2}-\varphi _{3} \vert + \vert \psi _{2}- \psi _{3} \vert , \vert \varphi _{3}- \varphi _{1} \vert + \vert \psi _{3}-\psi _{1} \vert \bigr\} \\ &\quad= \max \bigl\{ \vert \varphi _{\imath }-\varphi _{\jmath } \vert + \vert \psi _{\imath }- \psi _{\jmath } \vert : \imath,\jmath =1,2,3, \imath \neq \jmath \bigr\} \\ &\quad:=\mathfrak{B} \bigl((\varphi _{1},\psi _{1}),(\varphi _{2},\psi _{2}),( \varphi _{3},\psi _{3}) \bigr). \end{aligned}$$

Hence, the function $\mathfrak{B} ((\varphi _{1},\psi _{1}),(\varphi _{2},\psi _{2}),( \varphi _{3},\psi _{3}) )$ is a metric. □

This metric forms the maximum measurement between the three cases of growth of COVID-19. Note that this metric can be extended to include other cases in dynamic systems.

#### Theorem 3.3

*Suppose that the dynamic system* (2) *satisfies hypotheses* (*A*1) *and* (*A*2). *If the positive constantsκandKare such that*

$$\begin{aligned} \kappa < \frac{1}{1-\nu +\frac{T^{\nu }}{\varGamma (\nu )}} \quad\textit{and}\quad K< \frac{1}{1-\nu +\frac{T^{\nu }}{\varGamma (\nu )} },\quad T< \infty, \end{aligned}$$

*then*$\mathcal{P}$*has a unique fixed point in the ball*$B_{r}$, *where*$r \leq 1$.

#### Proof

In view of the assumption on *κ*, and the definition of the metric in Lemma 3.2, we have

$$\begin{aligned} & \mathfrak{B}\bigl(\mathcal{P}_{1}(\varphi _{1},\psi _{1}) (t), \mathcal{P}_{1} (\varphi _{2},\psi _{2}) (t),\mathcal{P}_{1}(\varphi _{3}, \psi _{3}) (t)\bigr) \\ &\quad= \max \bigl\{ \bigl\vert \mathcal{P}_{1}(\varphi _{\imath }, \psi _{\imath }) (t)- \mathcal{P}_{1}(\varphi _{\jmath },\psi _{\jmath }) (t) \bigr\vert : \imath,\jmath =1,2,3, \imath \neq \jmath \bigr\} \\ &\quad= \max \biggl\{ \biggl\vert (1-\nu ) X(t,\varphi _{\imath },\psi _{\imath })+ \frac{\nu }{\varGamma (\nu )} \int _{0}^{t}X(\tau,\varphi _{\imath },\psi _{\imath }) (t-\tau )^{\nu -1} \,d\tau \\ &\qquad{}-(1-\nu ) X(t,\varphi _{\jmath },\psi _{\jmath })- \frac{\nu }{\varGamma (\nu )} \int _{0}^{t}X(\tau,\varphi _{\jmath },\psi _{\jmath }) (t-\tau )^{\nu -1} \,d\tau \biggr\vert : \imath,\jmath =1,2,3, \imath \neq \jmath \biggr\} \\ &\quad \leq \max \biggl\{ (1-\nu )\kappa \bigl( \vert \varphi _{\imath }- \varphi _{\jmath } \vert + \vert \psi _{\imath }-\psi _{\jmath } \vert \bigr) + \frac{T^{\nu }}{\varGamma (\nu )} \kappa \bigl( \vert \varphi _{\imath }-\varphi _{\jmath } \vert + \vert \psi _{\imath }- \psi _{\jmath } \vert \bigr):\\ &\qquad \imath,\jmath =1,2,3, \imath \neq \jmath \biggr\} \\ &\quad= \max \biggl\{ \kappa \biggl(1-\nu +\frac{T^{\nu }}{\varGamma (\nu )}\biggr) \bigl( \vert \varphi _{\imath }-\varphi _{\jmath } \vert + \vert \psi _{\imath }-\psi _{\jmath } \vert \bigr): \imath,\jmath =1,2,3, \imath \neq \jmath \biggr\} \\ &\quad=\kappa \biggl(1-\nu +\frac{T^{\nu }}{\varGamma (\nu )}\biggr) \max \bigl\{ \bigl( \vert \varphi _{\imath }-\varphi _{\jmath } \vert + \vert \psi _{\imath }-\psi _{\jmath } \vert \bigr): \imath,\jmath =1,2,3, \imath \neq \jmath \bigr\} \\ &\quad:=r_{1} \mathfrak{B}\bigl((\varphi _{1},\psi _{1}), (\varphi _{2},\psi _{2}),( \varphi _{3},\psi _{3})\bigr),\quad r_{1}< 1. \end{aligned}$$

This proves the boundedness of the operator $\mathcal{P}_{1}$ in the unit ball $B_{r1} $ of radius $0 < r_{1}< 1$. Similarly for $\mathcal{P}_{2}$,

$$\begin{aligned} &\mathfrak{B}\bigl(\mathcal{P}_{2}(\varphi _{1},\psi _{1}) (t),\mathcal{P}_{2} (\varphi _{2},\psi _{2}) (t),\mathcal{P}_{2}(\varphi _{3},\psi _{3}) (t)\bigr) \\ &\quad \leq r_{2} \mathfrak{B}\bigl((\varphi _{1},\psi _{1}), (\varphi _{2},\psi _{2}),( \varphi _{3},\psi _{3})\bigr),\quad r_{2}< 1, \end{aligned}$$

which is bounded in the ball $B_{r2}, 0 < r_{2}<1 $. Combining the above conclusions, we obtain that the operator $\mathcal{P}=(\mathcal{P}_{1},\mathcal{P}_{2})$ is bounded in $B_{r}= (B_{r1}, B_{r2}) $.

We proceed to investigate other properties of operator $\mathcal{P}_{1}$. Let $t,\tau \in (0,T)$ be such that if $t>\tau $ then $\varphi (t)>\varphi (\tau )$ (increasing function). A simple calculation implies that

$$\begin{aligned} & \mathfrak{B} (\mathcal{P}_{1}(\varphi _{1}, \psi _{1}) (t), \mathcal{P}_{1}(\varphi _{2},\psi _{2}) (t),\mathcal{P}_{1}(\varphi _{3}, \psi _{3}) (t) \\ &\qquad{}-\bigl(\mathcal{P}_{1}(\varphi _{1}, \psi _{1}) (\tau ), \mathcal{P}_{1}(\varphi _{2}, \psi _{2}) (\tau ),\mathcal{P}_{1}(\chi _{3}, \psi _{3}) (\tau ) \bigr) \\ &\quad= \mathfrak{B} \bigl(\mathcal{P}_{1} \bigl(\varphi _{1}(t)-\varphi _{1}( \tau ), \psi _{1}(t)-\psi _{1}(\tau ) \bigr),\mathcal{P}_{1} \bigl( \varphi _{2}(t)-\varphi _{2}(\tau ), \psi _{2}(t)-\psi _{2}(\tau ) \bigr), \\ &\qquad \mathcal{P}_{1} \bigl(\varphi _{3}(t)-\varphi _{3}( \tau ),\psi _{3}(t)-\psi _{3}(\tau ) \bigr) \bigr) \\ &\quad= \mathfrak{B} \bigl(\mathcal{P}_{1}\bigl(\varphi _{1}(t- \tau ), \psi _{1}(t- \tau )\bigr),\mathcal{P}_{1}\bigl( \varphi _{2}(t-\tau ),\psi _{2}(t-\tau )\bigr), \mathcal{P}_{1}\bigl(\varphi _{3}(t-\tau ),\psi _{3}(t-\tau ) \bigr) \bigr) \\ &\quad\leq \mathfrak{B} \bigl(\mathcal{P}_{1}\bigl(\varphi _{1}(t), \psi _{1}(t)\bigr), \mathcal{P}_{1} \bigl(\varphi _{2}(t), \psi _{2}(t)\bigr), \mathcal{P}_{1}\bigl( \varphi _{3}(t),\psi _{3}(t) \bigr) \bigr) \\ &\quad=\mathfrak{B} \bigl(\mathcal{P}_{1}(\varphi _{1},\psi _{1}) (t), \mathcal{P}_{1} (\varphi _{2},\psi _{2}) (t),\mathcal{P}_{1}(\varphi _{3}, \psi _{3}) (t)\bigr) \\ &\quad\leq r_{1} \mathfrak{B}\bigl((\varphi _{1},\psi _{1}), (\varphi _{2}, \psi _{2}),(\varphi _{3},\psi _{3})\bigr). \end{aligned}$$

Thus, $\mathcal{P}_{1}$ is equicontinuous on $B_{r1}$. Similarly for $\mathcal{P}_{2}$,

$$\begin{aligned} & \mathfrak{B} (\mathcal{P}_{2}(\varphi _{1}, \psi _{1}) (t), \mathcal{P}_{2}(\varphi _{2},\psi _{2}) (t),\mathcal{P}_{2}(\varphi _{3}, \psi _{3}) (t) \\ &\qquad{}-\bigl(\mathcal{P}_{2}(\varphi _{1}, \psi _{1}) (\tau ), \mathcal{P}_{2}(\varphi _{2}, \psi _{2}) (\tau ),\mathcal{P}_{2}( \varphi _{3}, \psi _{3}) (\tau ) \bigr) \\ &\quad= \mathfrak{B} \bigl(\mathcal{P}_{2} \bigl(\varphi _{1}(t)-\varphi _{1}( \tau ), \psi _{1}(t)-\psi _{1}(\tau ) \bigr),\mathcal{P}_{2} \bigl( \varphi _{2}(t)-\varphi _{2}(\tau ), \psi _{2}(t)-\psi _{2}(\tau ) \bigr), \\ &\qquad \mathcal{P}_{2} \bigl(\varphi _{3}(t)-\varphi _{3}( \tau ),\psi _{3}(t)-\psi _{3}(\tau ) \bigr) \bigr) \\ &\quad\leq r_{2} \mathfrak{B}\bigl((\varphi _{1},\psi _{1}), (\varphi _{2}, \psi _{2}),(\varphi _{3},\psi _{3})\bigr). \end{aligned}$$

Thus, the integral operator $\mathcal{P}$ is equicontinuous on $B_{r}$.

Next, we check the continuity of the integral operator $\mathcal{P}\in B_{r}$. By assuming $\varphi _{l}(t)-\eta _{l}(t)=\xi _{l}(t)$, and $\psi _{l}(t)-\lambda _{l}(t)=\upsilon _{l}(t)$, $l=1,2,3$, we obtain

$$\begin{aligned} & \mathfrak{B} \bigl(\mathcal{P}_{1} \bigl(\varphi _{1}(t)- \eta _{1}(t), \psi _{l}(t)-\lambda _{l}(t) \bigr),\mathcal{P}_{1} \bigl( \varphi _{2}(t)-\eta _{2}(t), \psi _{2}(t)-\lambda _{2}(t) \bigr), \\ &\qquad\mathcal{P}_{1} \bigl(\varphi _{3}(t)-\eta _{3}(t),\psi _{3}(t)- \lambda _{3}(t) \bigr) \bigr) \\ &\quad= \mathfrak{B} \bigl( \mathcal{P}_{1} \bigl(\bigl(\xi _{1}(t),\upsilon _{1}(t)\bigr) \bigr),\mathcal{P}_{1} \bigl(\bigl(\xi _{2}(t),\upsilon _{2}(t)\bigr) \bigr), \mathcal{P}_{1} \bigl(\bigl(\xi _{3}(t),\upsilon _{3}(t)\bigr) \bigr) \bigr) \\ &\quad \leq \max \biggl\{ (1-\nu )\kappa \bigl( \vert \xi _{\imath }-\xi _{\jmath } \vert + \vert \upsilon _{\imath }-\upsilon _{\jmath } \vert \bigr) + \kappa \frac{T^{\nu }}{\varGamma (\nu )} \bigl( \vert \xi _{\imath }-\xi _{\jmath } \vert + \vert \upsilon _{\imath }- \upsilon _{\jmath } \vert \bigr):\\ &\qquad \imath,\jmath =1,2,3, \imath \neq \jmath \biggr\} \\ &\quad=\kappa \biggl(1-\nu +\frac{T^{\nu }}{\varGamma (\nu )}\biggr) \max \bigl\{ \bigl( \vert \xi _{\imath }-\xi _{\jmath } \vert + \vert \upsilon _{\imath }- \upsilon _{\jmath } \vert \bigr): \imath,\jmath =1,2,3, \imath \neq \jmath \bigr\} \\ &\quad=r_{1} \mathfrak{B}(\xi _{1},\upsilon _{1}), ( \xi _{2},\upsilon _{2}),( \xi _{3},\upsilon _{3})), \quad r_{1}< 1 \\ &\quad \leq r_{1} \mathfrak{B} \bigl((\varphi _{1},\psi _{1}),(\varphi _{2}, \psi _{2}),(\varphi _{3},\psi _{3}) \bigr). \end{aligned}$$

Therefore, the operator $\mathcal{P}_{1}$ is continuous in $B_{r1}$. Similarly, for $\mathcal{P}_{2}$, which leads to the conclusion that $\mathcal{P}$ has a fixed point $\mathcal{P}(\varphi,\psi )=(\varphi,\psi )$ corresponding to the solution of the dynamic system (2).

Next, we aim to check inequality (3). Suppose that there are two continuous and nondecreasing functions $\flat _{1},\wp _{1}\colon [0, \infty )\to [0,\infty )$ such that $\flat _{1}(t),\wp _{1}(t)>0$ for $t>0$ and $\flat _{1}(0)=\wp _{1}(0)=0$. Now, suppose that

$$\begin{aligned} \flat _{1}(\epsilon )=\epsilon /r_{1},\qquad \wp _{1}(\epsilon )= \frac{\epsilon (1-r_{1})}{r_{1}}. \end{aligned}$$

Then by the boundedness of $\mathcal{P}_{1}$, we conclude that

$$\begin{aligned} &\flat _{1}(\mathfrak{B} \mathcal{P}_{1} \bigl((\varphi _{1}, \psi _{1}),(\varphi _{1}, \psi _{1}),(\varphi _{i},\psi _{i}) \bigr) \\ &\quad = \mathfrak{B}\mathcal{P}_{1} \bigl((\varphi _{1},\psi _{1}),( \varphi _{1},\psi _{1}),(\varphi _{i},\psi _{i}) \bigr)/r_{1} \\ &\quad \leq \mathfrak{B} \bigl((\varphi _{1},\psi _{1}),( \varphi _{2},\psi _{2}),( \varphi _{3},\psi _{3}) \bigr) \\ &\quad\leq \mathfrak{B}\bigl((\varphi _{1},\psi _{1}),(\varphi _{1},\psi _{1}),( \varphi _{i},\psi _{i})\bigr)+\mathfrak{B}\bigl((\varphi _{2},\psi _{2}),( \varphi _{2},\psi _{2}),(\varphi _{j},\psi _{j})\bigr)\\ &\qquad{}+\mathfrak{B}\bigl(( \varphi _{3},\psi _{3}),(\varphi _{3},\psi _{3}),(\varphi _{k},\psi _{k})\bigr) \\ &\quad= \flat _{1} \bigl(\mathfrak{B}\bigl((\varphi _{1},\psi _{1}),(\varphi _{1}, \psi _{1}),(\varphi _{i},\psi _{i})\bigr) \bigr) \\ &\qquad{}-\wp _{1} \bigl(\mathfrak{B}\bigl((\varphi _{1},\psi _{1}),(\varphi _{1}, \psi _{1}),(\varphi _{i},\psi _{i})\bigr)+\mathfrak{B}\bigl((\varphi _{2},\psi _{2}),( \varphi _{2},\psi _{2}),(\varphi _{j},\psi _{j})\bigr)\\ &\qquad{}+ \mathfrak{B}\bigl(( \varphi _{3},\psi _{3}),(\varphi _{3},\psi _{3}),(\varphi _{k},\psi _{k})\bigr) \bigr) \\ &\quad\leq \flat _{1}\bigl(\mathfrak{B}\bigl((\varphi _{1},\psi _{1}),(\varphi _{1}, \psi _{1}),(\varphi _{i},\psi _{i})\bigr)\bigr)-\wp _{1}\bigl( \mathfrak{B}\bigl((\varphi _{1}, \psi _{1}),(\varphi _{1},\psi _{1}),(\varphi _{i},\psi _{i})\bigr)\bigr) \\ &\qquad{}+\min \bigl\{ \mathfrak{B}\bigl((\varphi _{2},\psi _{2}),(\varphi _{2}, \psi _{2}), \mathcal{P}_{1}(\varphi _{2},\psi _{2})\bigr), \mathfrak{B}\bigl(( \varphi _{2},\psi _{2}),(\chi _{2},\psi _{2}),\mathcal{P}_{1}( \varphi _{1},\psi _{1})\bigr), \\ &\qquad\mathfrak{B}\bigl((\varphi _{1},\psi _{1}),( \varphi _{1},\psi _{1}),\mathcal{P}_{1}(\varphi _{1},\psi _{1})\bigr), \\ &\qquad\mathfrak{B}\bigl((\varphi _{1},\psi _{1}),(\varphi _{1},\psi _{1}), \mathcal{P}_{1}(\varphi _{2},\psi _{2})\bigr) \bigr\} . \end{aligned}$$

Hence, this implies that inequality (3) holds. Similarly, for $\mathcal{P}_{2}$, which implies that the integral operator $\mathcal{P}$ has a unique fixed point lying in $B_{r}=(B_{r1},B_{r2})$, $r\leq 1$. □

By taking $X(t,\varphi,\psi ): = \alpha _{1}(t) \varphi (t)+ \alpha (t) \psi (t)$ and $A(t,\varphi,\psi ): =\beta _{1}(t) \psi (t)+\beta (t)\alpha (t)$ in Theorem 3.3, we have the following result:

#### Theorem 3.4

*Consider the dynamic system* (1). *If*$T^{\nu }\leq \nu, \nu \in (0,1] $*then it admits a unique fixed point in the ball*$B_{r}$, *where*$r=(\alpha _{\max },\beta _{\max })= ( \max \{\alpha _{1}(t), \alpha (t)\},\max \{\beta _{1}(t),\beta (t)\} ) $.

#### Proof

Define an operator $\mathcal{Q}: \mathbb{R} \times \mathbb{R} \rightarrow \mathbb{R} \times \mathbb{R}$ as follows:

6 $$\begin{aligned} &\bigl(\mathcal{Q}(\varphi,\psi ) \bigr) (t) \\ &\quad = \bigl(\mathcal{Q}_{1}( \varphi, \psi ), \mathcal{Q}_{2}( \varphi,\psi ) \bigr) (t) \\ &\quad = \biggl( (1-\nu ) \bigl(\alpha _{1}(t) \varphi (t)+\alpha (t) \psi (t)\bigr) \\ &\qquad{}+\frac{\nu }{\varGamma (\nu )} \int _{0}^{t}\bigl(\alpha _{1}(\tau ) \varphi (\tau )+ \alpha (\tau ) \psi (\tau )\bigr) (t-\tau )^{\nu -1}\,d\tau, \\ &\qquad (1-\nu ) \bigl(\beta _{1}(t)\psi (t)+\beta (t)\varphi (t)\bigr) \\ &\qquad{}+ \frac{\nu }{\varGamma (\nu )} \int _{0}^{t} \bigl(\beta _{1}(\tau ) \psi ( \tau )+ \beta (\tau )\varphi (\tau )\bigr) (t-\tau )^{\nu -1} \,d\tau \biggr), \\ & \mathfrak{B}\bigl(\mathcal{Q}_{1}(\varphi _{1},\psi _{1}) (t), \mathcal{Q}_{1} (\varphi _{2},\psi _{2}) (t),\mathcal{Q}_{1}(\varphi _{3}, \psi _{3}) (t)\bigr) \\ &\quad= \max \bigl\{ \bigl\vert \mathcal{Q}_{1}(\varphi _{\imath }, \psi _{\imath }) (t)- \mathcal{Q}_{1}(\varphi _{\jmath },\psi _{\jmath }) (t) \bigr\vert : \imath,\jmath =1,2,3, \imath \neq \jmath \bigr\} \\ &\quad= \max \biggl\{ \biggl\vert (1-\nu ) \bigl(\alpha _{1}(t) \varphi _{\imath }(t)+ \alpha (t) \psi _{\imath }(t)\bigr)\\ &\qquad{}+\frac{\nu }{\varGamma (\nu )} \int _{0}^{t}\bigl(\alpha _{1}( \tau ) \varphi _{\imath }(\tau )+ \alpha (\tau )\psi _{\imath }(\tau )\bigr) (t- \tau )^{\nu -1}\,d\tau \\ &\qquad{}-(1-\nu ) \bigl(\alpha _{1}(t) \varphi _{\jmath }(t)- \alpha (t) \psi _{\jmath }(t)\bigr) \\ &\qquad{}-\frac{\nu }{\varGamma (\nu )} \int _{0}^{t}\bigl(\alpha _{1}(\tau ) \varphi _{\jmath }(\tau )+ \alpha (\tau ) \psi _{\jmath }(\tau )\bigr) (t-\tau )^{ \nu -1}\,d\tau \biggr\vert : \imath,\jmath =1,2,3, \imath \neq \jmath \biggr\} \\ &\quad \leq (1-\nu ) \alpha _{\max } \bigl( \vert \varphi _{\imath }- \varphi _{\jmath } \vert + \vert \psi _{\imath }-\psi _{\jmath } \vert \bigr)+ \frac{T^{\nu }}{\varGamma (\nu )} \alpha _{\max } \bigl( \vert \varphi _{\imath }- \varphi _{\jmath } \vert + \vert \psi _{\imath }-\psi _{\jmath } \vert \bigr) \\ &\quad \leq (1-\nu ) \alpha _{\max } \bigl( \vert \varphi _{\imath }- \varphi _{\jmath } \vert + \vert \psi _{\imath }-\psi _{\jmath } \vert \bigr)+ \frac{\nu }{\varGamma (\nu )} \alpha _{\max } \bigl( \vert \varphi _{\imath }- \varphi _{\jmath } \vert + \vert \psi _{\imath }-\psi _{\jmath } \vert \bigr) \\ &\quad \leq (1-\nu ) \alpha _{\max } \bigl( \vert \varphi _{\imath }- \varphi _{\jmath } \vert + \vert \psi _{\imath }-\psi _{\jmath } \vert \bigr)+\nu \alpha _{\max } \bigl( \vert \varphi _{\imath }-\varphi _{\jmath } \vert + \vert \psi _{\imath }- \psi _{\jmath } \vert \bigr) \\ &\quad=\alpha _{\max } \bigl( \vert \varphi _{\imath }-\varphi _{\jmath } \vert + \vert \psi _{\imath }-\psi _{\jmath } \vert \bigr),\quad \nu \in (0,1) \\ &\quad= \alpha _{\max } \mathfrak{B}\bigl((\varphi _{1},\psi _{1}), (\varphi _{2}, \psi _{2}),(\varphi _{3},\psi _{3})\bigr). \end{aligned}$$

This yields the boundedness of the operator $\mathcal{Q}_{1}$ in the unit ball $B_{\alpha _{\max }}$. Similarly for $\mathcal{Q}_{2}$,

$$\begin{aligned} \mathfrak{B}\bigl(\mathcal{Q}_{2}(\varphi _{1},\psi _{1}) (t),\mathcal{Q}_{2} (\varphi _{2},\psi _{2}) (t),\mathcal{Q}_{2}(\varphi _{3},\psi _{3}) (t)\bigr) \leq \beta _{\max } \mathfrak{B}\bigl((\varphi _{1},\psi _{1}), (\varphi _{2}, \psi _{2}),(\varphi _{3},\psi _{3})\bigr), \end{aligned}$$

which is bounded in the ball $B_{\beta _{\max }}$. Combining the above conclusions, we obtain that the operator $\mathcal{Q}=(\mathcal{Q}_{1},\mathcal{Q}_{2})$ is bounded in $B_{r}= (B_{\alpha _{\max }}, B_{\beta _{\max }}) $.

We proceed to investigate other properties of operator $\mathcal{Q}_{1}$. Let $t,\tau \in (0,T)$ be such that if $t>\tau $ then $\varphi (t)>\varphi (\tau )$ (increasing function). A simple calculation implies that

$$\begin{aligned} & \mathfrak{B} (\mathcal{Q}_{1}(\varphi _{1}, \psi _{1}) (t), \mathcal{Q}_{1}(\varphi _{2},\psi _{2}) (t),\mathcal{Q}_{1}(\varphi _{3}, \psi _{3}) (t) \\ &\qquad{}-\bigl(\mathcal{Q}_{1}\varphi _{1}(\tau ),\mathcal{Q}_{1}( \varphi _{2},\psi _{2}) (\tau ),\mathcal{Q}_{1}(\varphi _{3},\psi _{3}) ( \tau ) \bigr) \\ &\quad= \mathfrak{B} \bigl(\mathcal{Q}_{1} \bigl(\varphi _{1}(t)-\varphi _{1}( \tau ), \psi _{1}(t)-\psi _{1}(\tau ) \bigr),\mathcal{Q}_{1} \bigl( \varphi _{2}(t)-\varphi _{2}(\tau ), \psi _{2}(t)-\psi _{2}(\tau ) \bigr), \\ &\qquad \mathcal{Q}_{1} \bigl(\varphi _{3}(t)-\varphi _{3}( \tau ),\psi _{3}(t)-\psi _{3}(\tau ) \bigr) \bigr) \\ &\quad= \mathfrak{B} \bigl(\mathcal{Q}_{1}\bigl(\varphi _{1}(t- \tau ), \psi _{1}(t- \tau )\bigr),\mathcal{Q}_{1}\bigl( \varphi _{2}(t-\tau ), \psi _{2}(t-\tau )\bigr), \mathcal{Q}_{1}\bigl(\varphi _{3}(t-\tau ),\psi _{3}(t-\tau ) \bigr) \bigr) \\ &\quad\leq \mathfrak{B} \bigl(\mathcal{P}_{1}\bigl(\varphi _{1}(t),\psi _{1}(t)\bigr), \mathcal{Q}_{1} \bigl(\varphi _{2}(t), \psi _{2}(t)\bigr), \mathcal{Q}_{1}\bigl( \varphi _{3}(t),\psi _{3}(t) \bigr) \bigr) \\ &\quad=\mathfrak{B} \bigl(\mathcal{Q}_{1}(\varphi _{1},\psi _{1}) (t), \mathcal{Q}_{1} (\varphi _{2},\psi _{2}) (t),\mathcal{Q}_{1}(\varphi _{3}, \psi _{3}) (t)\bigr) \\ &\quad\leq \alpha _{\mathrm{max}} \mathfrak{B}\bigl((\varphi _{1},\psi _{1}), (\varphi _{2}, \psi _{2}),(\varphi _{3},\psi _{3})\bigr). \end{aligned}$$

Thus, $\mathcal{Q}_{1}$ is equicontinuous on $B_{\alpha _{\max }}$. Similarly for $\mathcal{Q}_{2}$,

$$\begin{aligned} & \mathfrak{B} (\mathcal{Q}_{2}(\varphi _{1}, \psi _{1}) (t), \mathcal{Q}_{2}(\varphi _{2},\psi _{2}) (t),\mathcal{Q}_{2}(\varphi _{3}, \psi _{3}) (t) \\ &\qquad{}-\bigl(\mathcal{Q}_{2}(\varphi _{1}, \psi _{1}) (\tau ), \mathcal{Q}_{2}(\varphi _{2}, \psi _{2}) (\tau ),\mathcal{Q}_{2}( \varphi _{3}, \psi _{3}) (\tau ) \bigr) \\ &\quad= \mathfrak{B} \bigl(\mathcal{Q}_{2} \bigl(\varphi _{1}(t)-\varphi _{1}( \tau ), \psi _{1}(t)-\psi _{1}(\tau ) \bigr),\mathcal{Q}_{2} \bigl( \varphi _{2}(t)-\varphi _{2}(\tau ), \psi _{2}(t)-\psi _{2}(\tau ) \bigr), \\ &\qquad \mathcal{Q}_{2} \bigl(\varphi _{3}(t)-\varphi _{3}( \tau ),\psi _{3}(t)-\psi _{3}(\tau ) \bigr) \bigr) \\ &\quad\leq \beta _{\max } \mathfrak{B}\bigl((\varphi _{1},\psi _{1}), (\varphi _{2}, \psi _{2}),(\varphi _{3},\psi _{3})\bigr). \end{aligned}$$

Thus, the integral operator $\mathcal{Q}$ is equicontinuous on $B_{r}=(B_{\alpha _{\max }},B_{\beta _{\max }})$.

Next, we check the continuity of the integral operator $\mathcal{Q}\in B_{r}$. Now, by letting $\varphi _{j}(t)-\eta _{j}(t)=\xi _{j}(t)$ and $\psi _{j}(t)-\lambda _{j}(t)=\upsilon _{j}(t)$, $j=1,2,3$, we get

$$\begin{aligned} & \mathfrak{B} \bigl(\mathcal{Q}_{1} \bigl(\varphi _{1}(t)- \eta _{1}(t), \psi _{l}(t)-\lambda _{l}(t) \bigr),\mathcal{Q}_{1} \bigl( \varphi _{2}(t)-\eta _{2}(t), \psi _{2}(t)-\lambda _{2}(t) \bigr), \\ &\qquad\mathcal{Q}_{1} \bigl(\varphi _{3}(t)-\eta _{3}(t), \psi _{3}(t)- \lambda _{3}(t) \bigr) \bigr) \\ &\quad= \mathfrak{B} \bigl( \mathcal{Q}_{1} \bigl(\bigl(\xi _{1}(t),\upsilon _{1}(t)\bigr) \bigr),\mathcal{Q}_{1} \bigl(\bigl(\xi _{2}(t),\upsilon _{2}(t)\bigr) \bigr), \mathcal{Q}_{1} \bigl(\bigl(\xi _{3}(t),\upsilon _{3}(t)\bigr) \bigr) \bigr) \\ &\quad\leq \alpha _{\max } \mathfrak{B}\bigl((\varphi _{1},\psi _{1}), (\varphi _{2}, \psi _{2}),(\varphi _{3},\psi _{3})\bigr). \end{aligned}$$

Therefore, the operator $\mathcal{Q}_{1}$ is continuous in $B_{\alpha _{\max }}$. Similarly, for $\mathcal{Q}_{2}$, which leads to a conclusion that $\mathcal{Q}$ has a fixed point $\mathcal{Q}(\varphi,\psi )=(\varphi,\psi )$ corresponding to the solution of the dynamic system (2). Finally, condition (3) can be verified in the same manner as in Theorem 3.4, by assuming

$$\begin{aligned} \flat _{1}(\epsilon )=\epsilon /\alpha _{\max }, \qquad \wp _{1}( \epsilon )=\frac{\epsilon (1-\alpha _{\max })}{\alpha _{\max }}, \quad 0< \alpha _{\max }< 1. \end{aligned}$$

Thus, $\mathcal{Q}$ has a unique fixed point lying in $B_{\alpha _{\max },\beta _{\max }}=(B_{\alpha _{\max }},B_{\beta _{\max }})$. This completes the proof. □

### Approximate solvability

In this section, we consider a generalization for the Chebyshev polynomials of the first type by using the ABC operator. We shall present two cases. The first one uses constant connections, and is called the symmetric solution. This case represents the setting when both *φ* and *ψ* have the same number of infected and cured. While the second case considers the connections as functions with respect to *t*.

#### Symmetric solvability with constant connections

Define the expanded formula of the solution by

7 $$\begin{aligned} \varphi (t)= \sum_{n=0}^{\infty } \alpha _{n}T_{n}(t),\qquad \psi (t)= \sum _{n=0}^{\infty }\beta _{n}T_{n}(t), \end{aligned}$$

where $T_{n}(t)$ indicates the Chebyshev polynomials of the first kind such that $T_{0}(t)=1, T_{n+1}(t)= 2tT_{n}(t)-T_{n-1}(t), n\geq 1$. Chebyshev polynomials are of unlimited significance in various parts of mathematics, mainly approximation theory. The integrals of Chebyshev polynomials are (see [8])

8 $$\begin{aligned} \int T_{n}(t)= \frac{1}{2} \biggl( \frac{T_{n+1}(t)}{n+1} - \frac{T_{n-1}(t)}{n-1} \biggr),\quad n>1. \end{aligned}$$

The solution of (1) is given by the following construction:

9 $$\begin{aligned} &\bigl(\varphi (t),\psi (t)\bigr) \\ &\quad= \biggl( (1-\nu ) \bigl( \varphi (t)+\psi (t)\bigr)+\frac{\nu }{\varGamma (\nu )} \int _{0}^{t}\bigl( \varphi (\tau )+\psi (\tau ) \bigr) (t-\tau )^{\nu -1} \,d\tau, \\ &\qquad (1-\nu ) \bigl( \varphi (t)+\psi (t)\bigr)+ \frac{\nu }{\varGamma (\nu )} \int _{0}^{t}\bigl( \varphi (\tau )+\psi (\tau ) \bigr) (t- \tau )^{\nu -1} \,d\tau \biggr) \\ &\quad\approx 2^{\nu -1} \biggl( (1-\nu ) \bigl( \varphi (t)+\psi (t)\bigr)+ \frac{\nu }{\varGamma (\nu )} \int _{0}^{t}\bigl( \varphi (\tau )+\psi (\tau ) \bigr) \,d\tau, \\ &\qquad (1-\nu ) \bigl( \varphi (t)+\psi (t)\bigr)+ \frac{\nu }{\varGamma (\nu )} \int _{0}^{t}\bigl( \varphi (\tau )+\psi (\tau ) \bigr) \,d\tau \biggr). \end{aligned}$$

Now, by using the definition of *φ* in (7) and the integral formula of $T_{n}$ in (8), we have

10 $$\begin{aligned} \varphi (t)={}& 2^{\nu -1} \biggl((1-\nu ) \bigl( \varphi (t)+ \psi (t)\bigr)+\frac{\nu }{\varGamma (\nu )} \int _{0}^{t}\bigl( \varphi (\tau )+ \psi (\tau ) \bigr) \,d\tau \biggr) \\ = {}&2^{\nu -1} \Biggl((1-\nu ) \Biggl( \sum_{n=0}^{\infty } \alpha _{n}T_{n}(t)+ \sum_{n=0}^{\infty } \beta _{n}T_{n}(t)\Biggr) \\ &{}+\frac{\nu }{\varGamma (\nu )} \int _{0}^{t}\Biggl(\sum _{n=0}^{\infty }\alpha _{n}T_{n}(\tau )+\sum_{n=0}^{\infty }\beta _{n}T_{n}( \tau )\Biggr) \,d\tau \Biggr) \\ ={}& 2^{\nu -1} \Biggl((1-\nu ) \Biggl( \sum_{n=0}^{\infty } \alpha _{n}T_{n}(t)+ \sum_{n=0}^{\infty } \beta _{n}T_{n}(t)\Biggr) \\ &{}+\frac{\nu }{\varGamma (\nu )} \Biggl( \sum _{n=0}^{\infty }\alpha _{n} \int _{0}^{t}T_{n}(\tau )\,d\tau +\sum _{n=0}^{\infty }\beta _{n} \int _{0}^{t}T_{n}(\tau ) \,d\tau \Biggr) \Biggr) \\ \approx{}& 2^{\nu -1} (1-\nu ) \Biggl(\sum_{n=0}^{\infty } \alpha _{n}T_{n}(t)+ \sum_{n=0}^{\infty } \beta _{n}T_{n}(t) \Biggr) + \frac{ \nu }{2^{2-\nu }\varGamma (\nu )} \sum _{n=2}^{\infty }\alpha _{n} \biggl( \frac{T_{n+1}(t)}{n+1} - \frac{T_{n-1}(t)}{n-1} \biggr) \\ &{}+\frac{\nu }{2^{2-\nu }\varGamma (\nu )} \sum_{n=2}^{\infty }\beta _{n} \biggl( \frac{T_{n+1}(t)}{n+1} - \frac{T_{n-1}(t)}{n-1} \biggr). \end{aligned}$$

By symmetry, we obtain

11 $$\begin{aligned} \varphi (t)&=\psi (t) \\ &\approx 2^{\nu } (1-\nu ) \Biggl(\sum_{n=0}^{\infty } \alpha _{n}T_{n}(t) \Biggr) +\frac{ \nu }{2^{1-\nu }\varGamma (\nu )} \sum _{n=2}^{\infty }\alpha _{n} \biggl( \frac{T_{n+1}(t)}{n+1} - \frac{T_{n-1}(t)}{n-1} \biggr). \end{aligned}$$

By the assumption $t \leq t^{\nu }< T^{\nu }\leq \nu \leq 1$ (see Theorem 3.4), we have that the asymptotic behavior of the Chebyshev polynomials is

$$\begin{aligned} T_{n}(t) \sim 1,\quad \forall n, t\rightarrow 1. \end{aligned}$$

Thus, the finite case of (11) becomes

12 $$\begin{aligned} \varphi _{N}(t)&=\psi _{N}(t) \\ &\approx 2^{\nu } (1-\nu ) \Biggl(\sum_{n=0}^{N} \alpha _{n} \Biggr) + \frac{ \nu }{2^{1-\nu }\varGamma (\nu )} \sum _{n=2}^{N} \alpha _{n} \biggl( \frac{1}{n+1} - \frac{1}{n-1} \biggr). \end{aligned}$$

Consequently, by the convexity of the functions $\varphi (t)=\psi (t)$ which are majored by $\frac{1}{1-t}, t\in (0,1)$, we have the following construction:

13 $$\begin{aligned} \begin{aligned} & \alpha _{2}\leq \frac{1}{ (2^{\nu } (1-\nu )+ \frac{ \nu }{2^{1-\nu }\varGamma (\nu )} (-\frac{2}{3}) )}, \\ & \alpha _{3}\leq \frac{1}{ (2^{\nu } (1-\nu )+ \frac{ \nu }{2^{1-\nu }\varGamma (\nu )} (-\frac{1}{4}) )}, \\ & \alpha _{4} \leq \frac{1}{ (2^{\nu } (1-\nu )+ \frac{ \nu }{2^{1-\nu }\varGamma (\nu )} (-\frac{2}{15}) )}, \\ &\vdots \end{aligned} \end{aligned}$$

For example,

14 $$\begin{aligned} &\nu =0.1 \rightarrow \alpha _{2}=1.040, \alpha _{3}=1.038, \alpha _{4}=1.037, \dots, \\ &\nu =0.25 \rightarrow \alpha _{2}=1.156, \alpha _{3}=1.134, \alpha _{4}=1.128, \dots, \\ \begin{aligned} &\nu =0.5 \rightarrow \alpha _{2}=1.741, \alpha _{3}=1.521, \alpha _{4}=1.469, \dots, \\ &\nu =0.75 \rightarrow \alpha _{2}=12.929, \alpha _{3}=3.427, \alpha _{4}=2.842, \dots, \end{aligned} \\ &\nu =0.9 \rightarrow \alpha _{2}=-2.965, \alpha _{3}=-101.588, \alpha _{4}=12.220, \dots, \\ &\nu =1 \rightarrow \alpha _{2}=-3/2, \alpha _{3}=-4, \alpha _{4}=-15/2, \dots. \end{aligned}$$

Thus, the approximate symmetric solution can be seen as follows:

15 $$\begin{aligned} \begin{aligned}\varphi _{2}(t)={}& \alpha _{2} T_{2}(t) \approx \frac{2t^{2}-1}{ (2^{\nu } (1-\nu )+ \frac{ \nu }{2^{1-\nu }\varGamma (\nu )} (-\frac{2}{3}) )}, \\ \varphi _{3}(t)={}& \alpha _{2} T_{2}(t) +\alpha _{3} T_{3}(t) \approx \frac{2t^{2}-1}{ (2^{\nu } (1-\nu )+ \frac{ \nu }{2^{1-\nu }\varGamma (\nu )} (-\frac{2}{3}) )}+ \frac{4t^{3}-3t}{ (2^{\nu } (1-\nu )+ \frac{ \nu }{2^{1-\nu }\varGamma (\nu )} (-\frac{1}{4}) )}, \\ \varphi _{4}(t)= {}&\alpha _{2} T_{2}(t) +\alpha _{3} T_{3}(t) + \alpha _{4} T_{4}(t) \\ \approx{}& \frac{2t^{2}-1}{ (2^{\nu } (1-\nu )+ \frac{ \nu }{2^{1-\nu }\varGamma (\nu )} (-\frac{2}{3}) )}\\ &{}+ \frac{4t^{3}-3t}{ (2^{\nu } (1-\nu )+ \frac{ \nu }{2^{1-\nu }\varGamma (\nu )} (-\frac{1}{4}) )}+ \frac{8t^{4}-8t^{2}+1}{ (2^{\nu } (1-\nu )+ \frac{ \nu }{2^{1-\nu }\varGamma (\nu )} (-\frac{2}{15}) )}, \\ \vdots{}& \end{aligned} \end{aligned}$$

#### Symmetric solvability with functional connections

In this case, we have the following power series of the solution of (1):

16 $$\begin{aligned} \varphi (t)= \sum_{n=0}^{\infty } \alpha _{n}(t)T_{n}(t),\qquad \psi (t)= \sum _{n=0}^{\infty }\beta _{n}(t)T_{n}(t). \end{aligned}$$

The approximate solvability of (1) can be presented in the next result.

##### Theorem 3.5

*Consider system* (1) *with suitable nonconstant connections*$\alpha (t), \alpha _{1}(t), \beta (t)$, *and*$\beta _{1}(t)$*such that*$\delta (t):=\max_{t} \{\alpha (t), \alpha _{1}(t), \beta (t),\beta _{1}(t) \}$. *The approximate solution of* (1) *is*

$$\begin{aligned} \bigl(\varphi (t),\psi (t) \bigr) \approx \Biggl( \sum _{n=0}^{\infty }\delta _{\nu,n} ( 1+ C_{\nu,n} ) T_{n}(t), \sum_{n=0}^{\infty }\delta _{\nu,n} ( 1+ C_{\nu,n} ) T_{n}(t) \Biggr), \end{aligned}$$

*where*$\delta _{\nu,n}$*are constant coefficients and*

$$\begin{aligned} C_{\nu,n}= \frac{(-2)\varGamma (\nu +\frac{1}{2})}{c_{n}\varGamma (\frac{1}{2})\varGamma (\nu +1-n)\varGamma (\nu +1+n)}. \end{aligned}$$

##### Proof

From (1), we have the following solution for $\varphi (t)$ and similar conclusion for $\psi (t)$:

17 $$\begin{aligned} \varphi (t)&\approx \delta (t) (1-\nu ) \bigl( \varphi (t)+ \psi (t) \bigr)+\frac{\nu }{\varGamma (\nu )} \int _{0}^{t} \delta (\tau ) \bigl( \varphi (\tau )+ \psi (\tau )\bigr) (t-\tau )^{\nu -1} \,d\tau \\ &\approx \mu _{\nu } \biggl( f(t)+\frac{1}{\varGamma (\nu )} \int _{0}^{t} f( \tau ) (t-\tau )^{\nu -1} \,d \tau \biggr) \\ &= \mu _{\nu } \bigl( f(t)+ I^{\nu }f(t) \bigr),\quad \nu >0, t>0, \end{aligned}$$

where $\mu _{\nu }:=\max (\nu,1-\nu )$, $f(t)=\delta (t) ( \varphi (t)+\psi (t) ) $ and $I^{\nu }f(t)$ is the Riemann–Liouville integral operator. Assuming that

$$\begin{aligned} f(t)=\sum_{n=0}^{\infty }\delta _{n}(t) T_{n}(t), \end{aligned}$$

where (see [9])

18 $$\begin{aligned} \delta _{n}(t)&\approx \frac{1}{\hbar _{n}} \int _{0}^{1} f(t) T_{n}(t)\omega (t) \,dt \\ &:=\frac{2}{c_{n}\pi } \int _{0}^{1} f(t) T_{n}(t) \frac{1}{\sqrt{t-t^{2}}}\,dt \\ &\approx \frac{2}{\pi } \int _{0}^{1} f(t) \,dt \\ &:=\delta _{n},\quad \forall n, \end{aligned}$$

where the parameters satisfy $t< T^{\nu }<\nu <1$ (see Theorem 3.4), and $c_{n}=1$, $c_{0}=2$, $T_{n}\sim 1$. Moreover, in view of Theorem 3.1 [9], we have

19 $$\begin{aligned} \varphi (t)&\approx \delta (t) (1-\nu ) \bigl( \varphi (t)+ \psi (t) \bigr)+\frac{\nu }{\varGamma (\nu )} \int _{0}^{t} \delta (\tau ) \bigl( \varphi (\tau )+ \psi (\tau )\bigr) (t-\tau )^{\nu -1} \,d\tau \\ &\approx \mu _{\nu } \biggl( f(t)+\frac{1}{\varGamma (\nu )} \int _{0}^{t} f( \tau ) (t-\tau )^{\nu -1} \,d \tau \biggr) \\ &= \mu _{\nu } \bigl( f(t)+ I^{\nu }f(t) \bigr), \quad\nu >0, t>0, \\ &=\mu _{\nu } \Biggl( \sum_{n=0}^{\infty } \delta _{n} T_{n}(t)+ \sum_{n=0}^{\infty } \delta _{n} C_{\nu,n} T_{n}(t) \Biggr) \\ &=\mu _{\nu }\sum_{n=0}^{\infty }\delta _{n} T_{n}(t) ( 1+ C_{\nu,n} ) \\ &:= \sum_{n=0}^{\infty }\delta _{\nu,n} ( 1+ C_{\nu,n} ) T_{n}(t), \end{aligned}$$

where

$$\begin{aligned} C_{\nu,n}:= \frac{(-2)\varGamma (\nu +\frac{1}{2})}{c_{n}\varGamma (\frac{1}{2})\varGamma (\nu +1-n)\varGamma (\nu +1+n)}. \end{aligned}$$

This completes the proof. □

Note that

$$\begin{aligned} \delta _{\nu,n} \approx \textstyle\begin{cases} \frac{1}{\pi } & \text{if }\nu \geq 1/2, \\ \frac{2}{\pi } & \text{if }\nu < 1/2. \end{cases}\displaystyle \end{aligned}$$

And

$$\begin{aligned} &\nu =0.5, n=2 \rightarrow C_{\nu,n}=0.0957,\qquad \nu =0.75, n=2 \rightarrow C_{\nu,n}=0.0471, \\ &\nu =0.5, n=3 \rightarrow C_{\nu,n}=-0.0410, \qquad\nu =0.75, n=3 \rightarrow C_{\nu,n}=-0.0157, \\ &\nu =0.5, n=4 \rightarrow C_{\nu,n}=0.0228, \qquad\nu =0.75, n=4 \rightarrow C_{\nu,n}=0.0074, \\ &\nu =0.5, n=5 \rightarrow C_{\nu,n}=-0.0145,\qquad \nu =0.75, n=5 \rightarrow C_{\nu,n}=-0.0042. \end{aligned}$$

It is clear that $|C_{\nu,n}|<1$ for all $n\geq 2$ and $\nu \in [0,1]$. Therefore, for the finite case, the approximate solution can be evaluated as follows:

20 $$\begin{aligned} \begin{aligned} &\varphi _{0}(t) \approx \frac{2}{\pi }T_{0}(t)= \frac{2}{\pi }, \qquad \nu \geq 0.5, \quad\text{or}\\ & \varphi _{0}(t)\approx \frac{4}{\pi },\qquad \nu < 0.5, \\ &\varphi _{1}(t) \approx \frac{2}{\pi }\bigl(T_{0}(t)+T_{1}(t) \bigr)= \frac{2t+2}{\pi }, \qquad \nu \geq 0.5, \quad\text{or}\\ & \varphi _{1}(t) \approx \frac{4t+4}{\pi },\qquad \nu < 0.5, \\ &\varphi _{2}(t) \approx \frac{2}{\pi }\bigl(T_{0}(t)+T_{1}(t)+T_{2}(t) \bigr)= \frac{2(t+2t^{2})}{\pi },\qquad \nu \geq 0.5,\quad \text{or}\\ & \varphi _{2}(t) \approx \frac{4(t+2t^{2})}{\pi },\qquad \nu < 0.5, \\ &\vdots \end{aligned} \end{aligned}$$

and similarly for $\psi (t)$.

## Application

**Figure 1:**
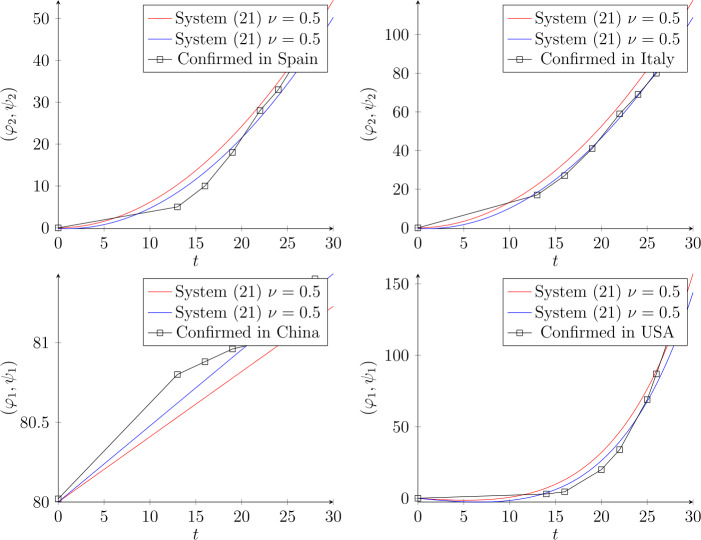
The dynamic evolution of system (21) when $\nu =0.5$, with the approximate solution by fractional Chebyshev polynomials $(\varphi _{2},\psi _{2})= (\frac{2(t+2t^{2})}{\pi }, \frac{2(t+2t^{2})}{\pi })$ for Spain and for Italy with different coefficients $C_{0.5,2} $. China statistics in March has steady circulation; consequently, we propose $(\varphi _{1},\psi _{1})= (\frac{2t+2}{\pi },\frac{2t+2}{\pi })$. For USA data, the chart shows high rising confirmed cases, therefore we apply exponential connections $\varphi _{1}(t)(\exp (\sqrt{\varphi _{1}(t)})-1)$ similarly for $\psi (t)$. Note that the data are shown in March

As an application, we assess our scheme by fitting real statistics from the Internet. Figure 1 illustrates the imitated data in March for the worst affected countries. We consider the following dynamic system:

21 $$\begin{aligned} \begin{aligned} &\Delta ^{0.5}\varphi (t) = \alpha _{1}(t) \varphi (t)+ \alpha (t) \psi (t), \\ & \Delta ^{0.5}\psi (t) =\beta _{1}(t) \psi (t)+\beta (t) \varphi (t). \end{aligned} \end{aligned}$$

**Figure 2:**
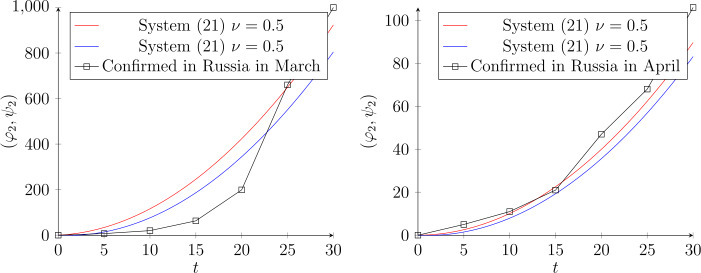
The dynamic evolution of system (21) when $\nu =0.5$, with the approximate solution by fractional Chebyshev polynomials $(\varphi _{2},\psi _{2})= (\frac{2(t+2t^{2})}{\pi }, \frac{2(t+2t^{2})}{\pi })$ for Russia in March and April, respectively. In March the number of infections was per person, while in April it was per K (thousand). The connection coefficient in March is $C_{0.5,2} =0. 79$, while in April it is $C_{0.5,2} =0. 099$

**Figure 3:**
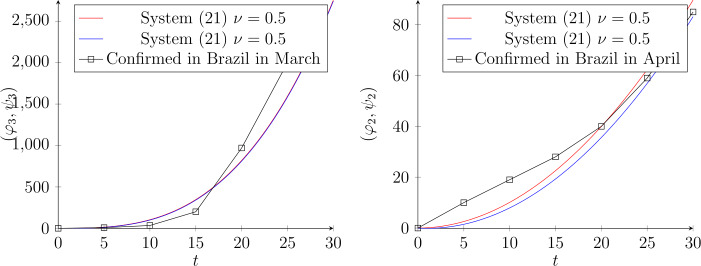
The dynamic evolution of system (21) when $\nu =0.5$, for Brazil in March and April, respectively. In March the number of infections was per person, therefore, we used $(\varphi _{3},\psi _{3})$. In April the data was per $K=1000$, therefore, we find that $(\varphi _{2},\psi _{2})$ is a suitable solution

By employing different approximations (20), in Fig. 1 we plot them depending on the statistics of the data. The approximate solution of (21), in the case of Spain and Italy, is $(\varphi _{2},\psi _{2})= (\frac{2(t+2t^{2})}{\pi }, \frac{2(t+2t^{2})}{\pi })$ for the connection constants $C_{0.5,2} =0. 0957$ and $C_{0.5,2} =0. 0471$, respectively. While the data of China indicate using $(\varphi _{1},\psi _{1})= (\frac{2t+2}{\pi },\frac{2t+2}{\pi })$ with the maximum value of connection $C_{0.5,1} =1 $. The information about USA recognizes rapidly increasing cases, therefore, we used the combined function of $\varphi _{1}$ as follows: $\varphi _{1}(t)(\exp (\sqrt{\varphi _{1}(t)})-1)$ with connection value $C_{0.5,1} =0. 023 $. Note that the confirmed cases are measured in thousands, for example, in Spain, the number of confirmed cases in March was 95.9 K, while in April it was 236.899 K, therefore, the approximate solution is given by $(\varphi _{4},\psi _{4})$. Figure 2 indicates the cases in Russia in March and April. The data show that in March the number of infections was very low (per person), but in April it was increasing rapidly (per $K=1000$) but it is still approximated by $(\varphi _{2},\psi _{2})$. We confirmed that the approximate solution by fractional Chebyshev polynomials fits the future expectation of the number of infections. We added also the case of Brazil. The picture is similar also for Brazil data, which indicates huge changes from March to April (see Fig. 3).

## Conclusion

Based on the above, we conclude that the fractional-fractal dynamic system based on the Atangana–Baleanu fractional operator indicates flexibility and accuracy of introducing approximate solutions by fractional Chebyshev polynomials. For our future work, we aim to use the same calculus (ABC) to generalize different polynomials to get an optimal solution.
